# Explaining the effects of a 1-year intervention promoting a low fat diet in adolescent girls: a mediation analysis

**DOI:** 10.1186/1479-5868-4-55

**Published:** 2007-11-09

**Authors:** Leen Haerens, Ester Cerin, Benedicte Deforche, Lea Maes, Ilse De Bourdeaudhuij

**Affiliations:** 1 Ghent University, Department of Movement and Sports Sciences, Watersportlaan 2, Gent, 9000, Belgium; 2 University of Hong Kong, Institute of Human Performance, 111 Pokfulam Road, Pokfulam, Hong Kong; 3 Ghent University, Department of Public Health, De Pintelaan 185, BLOK A, Gent, 9000, Belgium

## Abstract

**Background:**

Although it is important to investigate how interventions work, no formal mediation analyses have been conducted to explain behavioral outcomes in school-based fat intake interventions in adolescents. The aim of the present study was to examine mediation effects of changes in psychosocial determinants of dietary fat intake (attitude, social support, self-efficacy, perceived benefits and barriers) on changes in fat intake in adolescent girls.

**Methods:**

Data from a 1-year prospective intervention study were used. A random sample of 804 adolescent girls was included in the study. Girls in the intervention group (n = 415) were exposed to a multi-component school-based intervention program, combining environmental changes with a computer tailored fat intake intervention and parental support. Fat intake and psychosocial determinants of fat intake were measured with validated self-administered questionnaires. To assess mediating effects, a product-of-coefficient test, appropriate for cluster randomized controlled trials, was used.

**Results:**

None of the examined psychosocial factors showed a reliable mediating effect on changes in fat intake. The single-mediator model revealed a statistically significant suppression effect of perceived barriers on changes in fat intake (*p *= 0.011). In the multiple-mediator model, this effect was no longer significant, which was most likely due to changes in perceived barriers being moderately related to changes in self-efficacy (-0.30) and attitude (-0.25). The overall mediated-suppressed effect of the examined psychosocial factors was virtually zero (total mediated effect = 0.001; SE = 7.22; *p *= 0.992).

**Conclusion:**

Given the lack of intervention effects on attitudes, social support, self-efficacy and perceived benefits and barriers, it is suggested that future interventions should focus on the identification of effective strategies for changing these theoretical mediators in the desired direction. Alternatively, it could be argued that these constructs need not be targeted in interventions aimed at adolescents, as they may not be responsible for the intervention effects on fat intake. To draw any conclusions regarding mediators of fat-intake change in adolescent' girls and regarding optimal future intervention strategies, more systematic research on the mediating properties of psychosocial variables is needed.

## Background

Dietary behavior interventions typically aim at influencing a set of implicit or explicit mediating variables derived from social-psychological theories such as the *theory of planned behaviour *[1], *the social cognitive theory *[2] and *the attitude, social influence and self-efficacy model *[3]. These theories postulate that an intervention can change behavior through changing one or more theoretical antecedents or determinants of the behavior of interest [4]. Despite these assumptions, only a few formal mediation analyses have been thus far conducted to explore mechanisms of change in school-based nutrition intervention programs. Reynolds et al [5] identified 'positive outcome expectations' as a mediating variable in a school-based nutrition intervention designed to increase fruit and vegetable consumption in 4^th ^graders. In contrast, self-efficacy, parent consumption and knowledge were not found to be significant mediators. In another study, knowledge was identified as a mediator of fruit and vegetable intake in 4^th ^graders, while parental support and availability were not [6].

To our knowledge, no mediation analyses of effective dietary interventions for adolescents have been thus far conducted. One reason for the lack of studies investigating mediating variables is that, to date, only few school-based interventions tried to target dietary behavior in adolescents. Furthermore, a proper mediation analysis requires a control group and a prospective design allowing an examination of whether changes in the hypothesized mediators affect changes in the outcome. A Belgian intervention study in middle schools met these requirements [7]. Hence, the data of this study provided an opportunity to identify mediating variables responsible for improving dietary behaviors in adolescents.

The aim of the present study was to examine the mediating effects of changes in psychosocial determinants of dietary fat intake (attitude, social support, self-efficacy, perceived benefits, and barriers) on changes in fat intake in adolescent girls, using a 1-year prospective intervention study. As the intervention was developed to change all these underlying constructs, it was hypothesized that changes in these constructs would mediate changes in fat intake from baseline to one year follow-up.

## Methods

### Procedure and participants

A random sample of 15 out of the 65 Flemish schools with technical and vocational education in West-Flanders (Belgium) was selected to participate in the intervention study [7]. These 15 schools were then randomly assigned to the intervention or control conditions (5 schools per condition): (a) Intervention with parental support, (b) intervention alone and (c) control condition. The parents of all 2991 pupils in 7^th ^and 8^th ^grade received an informed consent form seeking their authorization for their child to take part in the study. The parents of 151 (5%) children did not give permission for their child to participate in this study. This resulted in a sample of 2840 11-to-15 year old boys and girls within 15 schools. A more detailed description of the sample and procedure was reported elsewhere [7]. For the present study, data from girls of the intervention group with parental support and the control group were included. This is because previous analyses revealed that only the intervention with parental support was effective in changing fat intake, and only among girls [7]. Of the 843 girls participating at baseline, 788 participated at follow-up (93.5%). Data were missing for 55 girls due to them not attending the post-test measurements or inadequately filling-out the questionnaires.

The Study protocol was approved by the Ethical Committee of Ghent University.

### Measures

Measures were taken at school, during class hours and under the direct supervision of teachers, at the beginning (September 2003) and at the end of the school year (June 2004).

### Food frequency questionnaires

Fat intake was measured with a self-administered questionnaire developed at the Ghent University together with the Flemish Institute for Health Promotion [8]. The questionnaire was validated in a separate study and was found to be sufficiently reliable (ICC = 0.86) and valid as compared to a 7-day dietary record method (Pearson r = 0.67) [8]. The questionnaire consisted of 48 items, representing all important sources of fat in the Belgian diet. Pupils were asked how often they consumed these products during a usual day, week or month. A coefficient was calculated, representing the fat content and portion size of each product. This coefficient was multiplied by the frequency of consumption, leading to a fat intake score for each food item.

### Psychosocial determinants

The food frequency questionnaire ended with a three-page long survey asking about psychosocial determinants of a low fat diet. The questions were similar to those used in previous studies [9]. Based on the Social Cognitive Theory [1] and the Theory of Planned Behavior [2] the following groups of determinants were included in the study: attitude (4 items; Cronbach's α = 0.83), self-efficacy (2 items; Cronbach's α = 0.38), social support (4 items; Cronbach's α = 0.71), perceived benefits (6 items; Cronbach's α = 0.83), and perceived barriers (12 items Cronbach's α = 0.85).

Attitudes towards a low fat diet were rated on a five-point scale from "certainly not pleasant" (good/tasty/healthy) "to certainly pleasant" (good/tasty/healthy). To measure self-efficacy, pupils were asked to rate on a five-point scale how difficult it was to eat a low fat diet at home or at school. To measure social support, pupils were asked to rate on a five-point scale how frequently significant others (parents, brothers and sisters, friends, and teachers) supported their eating a low fat diet. For perceived benefits (e.g. health, taste, losing weight) and barriers (e.g. lack of time, not available, not accessible, expensive) a five-point scale ranging from "totally disagree" to "totally agree" was used.

### Intervention

The intervention was designed to be implemented by the school staff with only minimal external support to make later implementation more feasible. It was coordinated by a working group of school personnel that received background information, an intervention manual and educational material from the researchers. The healthy eating intervention had an environmental and an individual-based component. The environmental intervention focused on increasing availability of healthy products, such as water and fruit, and decreasing the availability of unhealthy products (e.g., soft drinks). At the personal level, students completed a youth-based version of the computer **-t**ailored fat intake intervention [7,10] during one class hour. At the end of the intervention, they were given immediate personal feedback about their fat intake. The Transtheoretical Model [11] was used to define the content and approach of the feedback. Based on the theory of Planned Behavior [2] children received tailored feedback about their attitudes, self-efficacy, social support, knowledge, benefits and barriers related to their fat intake. A healthy diet was promoted using a 5–6 page written advice.

In the intervention group with parental support, a CD with the adult computer-tailored intervention for fat intake [10] was given to all parents for them to use and complete at home. Parents were thus made aware of their own fat intake and its consequences for health. The advice gave personalized information on how to make changes towards a more healthy diet. Parents were informed in writing that their child had been exposed to a similar computer-tailored tool in class. They were asked to discuss with their child the feedback that they both received. They were also asked to support their child in adopting the healthy changes suggested in the feedback.

A more thorough description of the dietary intervention is reported elsewhere [7].

### Statistical Analyses

Preliminary analyses consisted of descriptive statistics of sample characteristics. Independent sample t-tests and χ^2 ^tests were used to conduct drop-out analyses (see Table 1).

**Table 1:** Descriptive Characteristics (percentages or means and standard deviations) for Baseline Sample and Follow-up Sample

	Baseline sample *(n = 843)*	Follow-up sample *(n = 788)*	Drop out *(n = 55)*	
	*M *± *SD*	*M *± *SD*	*M *± *SD*	χ^2^
% Higher SES	39.5	40.0	32.7	1.1
				*F*_drop out_
Age (years)	12.9 ± 0.8	12.9 ± 0.8	13.0 ± 0.9	-0.1
Fat intake (g/day)	98.2 ± 39.0	97.8 ± 38.9	103.6 ± 41.1	-1.1
Attitude	3.5 ± 0.6	3.5 ± 0.6	3.3 ± 0.6	2.5**
Self-efficacy	3.5 ± 0.8	3.5 ± 0.8	3.5 ± 0.8	0.4
Social support	2.0 ± 0.7	2.0 ± 0.7	2.1 ± 0.6	-0.3
Perceived benefits	3.1 ± 0.8	3.1 ± 0.8	3.1 ± 0.8	0.6
Perceived barriers	2.2 ± 0.7	2.2 ± 0.7	2.3 ± 0.7	-1.4

**p ≤ 0.01

Changes in attitudes, social support, self-efficacy, perceived benefits and perceived barriers to a low fat diet were examined as potential mediators of the intervention effect on fat intake in adolescent girls.

A measure of *change of fat intake *between pre- and post-test, free of autocorrelated error, was created by regressing the fat intake measures at post-test onto the fat intake measures at baseline to compute the residualized fat intake change score (the difference between the predicted and observed fat intake at posttest). The resulting residualized scores can be interpreted as the amount of increase or decrease in fat intake between baseline and posttest, independent of baseline fat intake. This approach to the operationalisation of changes in the outcome addresses problems related to regression to the mean (i.e. the fact that those with extreme outcome values at one point in time will tend to have less extreme values at the following assessment), and accounts for the possibility that those with high fat intake at baseline will have more room for change compared to those with low baseline fat intake [12]. Similarly, a measure of *change of psychosocial determinants *was recreated by regressing each psychosocial determinant score at posttest onto the baseline scores. Table 2 presents the baseline behavioral and psychosocial characteristics of the sample as well as the residual change scores for these variables.

**Table 2:** Baseline fat intake and psychosocial determinants, and residual change scores for the same variables by experimental condition

	Condition	Pre	Residual changes score
	*(n)*	(Mean ± SD)	(Mean ± SD)
***Fat intake (g/day)***	I *(415)*	97.05 ± 38.50	-15.12 ± 31.58
	C *(388)*	97.80 ± 38.97	-6.47 ± 30.95
Attitude	I *(415)*	3.55 ± 0.61	0.01 ± 0.54
	C *(388)*	3.47 ± 0.58	0.02 ± 0.52
Self-efficacy	I *(415)*	3.48 ± 0.80	0.01 ± 0.75
	C *(388)*	3.53 ± 0.77	0.06 ± 0.75
Social support	I *(415)*	2.07 ± 0.74	0.03 ± 0.62
	C *(388)*	1.98 ± 0.68	-0.02 ± 0.72
Perceived benefits	I *(415)*	3.18 ± 0.85	0.05 ± 0.76
	C *(388)*	3.12 ± 0.79	-0.02 ± 0.74
Perceived barriers	I *(415)*	2.15 ± 0.69	0.03 ± 0.63
	C *(388)*	2.27 ± 0.67	-0.07 ± 0.65

I: Intervention, C: Control group

To assess mediating effects, a product-of-coefficient test, appropriate for cluster randomized controlled trials, was used [13]. This tests consists of (1) estimating the effect of the intervention on changes in the potential mediator (α coefficient) by regressing changes in the mediator onto the intervention; (2) estimating the independent effect of changes in the potential mediator on changes in the outcome (β coefficient) by regressing changes in the outcome onto the intervention and changes in the mediator; (3) computing the product of the two coefficients αβ, representing the mediated effect; (4) dividing αβ by its standard error. The first (action theory test) and second (conceptual theory test) step in the analysis are identical to the second and third step in the Baron-Kenny causal step approach resulting in α and β coefficients, respectively. The estimates were obtained using two-level linear regression models, accounting for within-school cluster effects. As the outcome variable was skewed, Huber/White robust estimates of standard errors were used.

Although this intervention was meant to simultaneously target multiple mediators, both single- and multiple-mediator models were assessed [14], the reason being that the effect of a specific mediator in a multiple-mediator model may be obscured by the presence of multicollinearity [15]. Finally, the magnitude of the total mediated effect and ratios of mediated to total intervention effects were also estimated. The standard error of the total mediated effect was computed using the multivariate delta method, i.e., by pre- and post-multiplying the covariance matrix among α and β parameters of the function (sum of five mediating effects) by a vector of partial derivatives of the function [16]. Mediating variable analyses were conducted using MLwiN version 2.02 and Microsoft Excel.

## Results

### Power analyses

The study design effect for residualized changes in social support was 5.19 (effective sample size = 152), while that for residualized changes in self-efficacy was 2.02 (effective sample size 389). The design effect for the remaining variables was 1 (corresponding to no cluster effect). This means that, adopting a significance level of 0.05, the power of the study to detect small mediation effects (defined as a standardized change of 0.14; [17]) was approximately 0.32 for social support, 0.69 for perceived benefits and 0.95 for the other psychosocial variables. The study had 0.99 power to detect mediation effects of moderate (standardized change of 0.39) and large size (standardized change of 0.59).

### Sample characteristics and drop-out analysis

Baseline demographic and behavioral characteristics of the baseline and follow-up sample are shown in Table 1. Drop out analyses comparing baseline demographic and behavioral characteristics of adolescents participating and not participating at follow-up yielded one significant difference. Namely, adolescents who did not participate at follow-up had a less favorable attitude towards eating a low fat diet.

### Mediation analyses

Table 3 reports the results of the mediating variable analyses.

**Table 3:** Mediation effects of changes in psychosocial factors on changes in fat intake

Psychosocial factor	Single-mediator models					
	α(SE)	β(SE)	αβ(SE)	95% CI of αβ	z	% mediated effect
Attitude	-0.004 (0.031)	-7.773 (2.077)***	0.031 (0.241)	-0.441, 0.503	0.129	statistical suppression^a^
Self-efficacy	-0.020 (0.077)	-1.790 (1.581)	0.036 (0.139)	-0.236, 0.308	0.258	statistical suppression^a^
Social support	0.005 (0.098)	-2.548 (1.732)	-0.013 (0.250)	-0.502, 0.477	-0.051	0.1
Perceived benefits	0.069 (0.039)	-1.799 (2.997)	-0.124 (0.113)	-0.345, 0.097	-1.099	1.4
Perceived barriers	0.067 (0.022)**	2.260 (1.493)	0.151 (0.060)*	0.034, 0.268	2.535	suppression

	Multiple-mediator model					
	α(SE)	β(SE)	αβ(SE)	95% CI of αβ	z	% mediated effect

Attitude	-0.004 (0.031)	-6.966 (1.866)***	0.028 (0.054)	-0.078, 0.134	0.515	statistical suppression^a^
Self-efficacy	-0.020 (0.077)	-0.379 (1.941)	0.008 (0.040)	-0.070, 0.085	0.191	statistical suppression^a^
Social support	0.005 (0.098)	-1.643 (2.177)	-0.008 (0.037)	-0.080, 0.060	-0.223	0.1
Perceived benefits	0.069 (0.039)	-0.144 (3.055)	-0.010 (0.211)	-0.423, 0.403	-0.047	0.1
Perceived barriers	0.067 (0.022)**	0.786 (1.741)	0.053 (0.117)	-0.177, 0.282	0.450	statistical suppression^a^

α estimate of intervention effect (unstandardized regression coefficient) on residualized change score of psychosocial factors

β estimate of the independent effect of the mediator (unstandardized regression coefficient of residualized change scores) on residualized change score for fat intake αβ product-of-coefficient estimate; mediated effect

SE standard error

95% CI of αβ 95% confidence interval of the mediated effect

z standard deviate associated with mediated effect (used for significance testing)

* significant at the 5% probability level, ** significant at the 1% probability level; *** significant at the 0.1% probability level

a Statistical suppression refers to a statistically non-significant indirect effect that is opposite in sign to the intervention effect

### Intervention effect

On average, the intervention group reduced their fat intake by 9.0 g/day (95% CI: -12.4, -5.6) more than did the control group, which corresponds to a medium effect size Cohen's *d *of 0.41 [17].

### Action Theory test (α coefficients; Table 3)

The intervention did not lead to significant positive changes in the psychosocial determinants. When compared to the control group, the intervention even appeared to have a negative effect on changes in perceived barriers (p < .01).

### Conceptual Theory test (β coefficients; Table 3)

Change in attitudes was the only determinant significantly associated with changes in dietary fat intake; changes were in the expected direction (p < .001; Table 3).

### Mediated effects

None of the examined psychosocial factors showed a reliable mediating effect on changes in fat intake (see Table 3). The single-mediator model revealed a statistically significant suppression effect of perceived barriers on changes in fat intake (*p *= 0.011). This was due to the intervention having a negative effect on this particular psychosocial factor (α = 0.067; SE = 0.022; *p *= 0.002). The average size of this suppression effect was approximately 0.15 g of fat intake per day, meaning that the intervention could have been more effective by 0.15 g/day (95% CI: 0.03, 0.27) if it had not lead to an increase in perceived barriers. In the multiple-mediator model, this effect was no longer significant, which was most likely due to changes in perceived barriers being moderately related to changes in self-efficacy (-0.30) and attitude (-0.25). The overall mediated-suppressed effect of the examined psychosocial factors was virtually zero (total mediated effect = 0.001; SE = 7.22; *p *= 0.992).

## Discussion

The aim of the present study was to investigate the mechanisms through which a school-based fat intake intervention in adolescent girls was effective. Mediation analyses of change in psychosocial determinants (attitude, social support, self-efficacy, perceived benefits and barriers) on change in fat intake over a 1 year period were used to derive this information. The findings revealed that the intervention did not change any of the hypothesized psychosocial mediators. In contrast to expectations, it even had a negative effect on perceived barriers.

Adolescent girls were exposed to an intervention consisting of three main components: 1) an environmental component mainly focusing on increasing availability of healthy products and decreasing availability of unhealthy products in the school environment, 2) an individual component based on personal feedback through online computer tailoring and 3) a parental component focusing on increasing support for healthy eating behaviors outside school.

The computer-tailoring part of the intervention included specific feedback on all psychosocial determinants. The environmental and parental components were also hypothesized to exert a positive impact on the psychosocial determinants. The increasing availability of healthy products in the school environment was, for example, assumed to have lead to increased self-efficacy for eating low fat products at school. Increasing parental support was, for example, hypothesized to lead to increased social support for a low fat diet. In contrast to these assumptions, the intervention effect on fat intake was not significantly mediated by attitudes, self-efficacy, social support, perceived benefits and perceived barriers. Even an unexpected suppressor effect was found for perceived barriers towards a low fat diet. There are five possible explanations for the lack of mediation effects. Firstly, although psychosocial measures were shown to be reliable and valid in cross-sectional research, they might have not been sufficiently sensitive to detect changes in these constructs. A second explanation regards the possibility that the intervention was not adequately implemented, which could have led to a lack of impact on the mediators. However, the positive effects on dietary fat intake suggest that the program was at least partly implemented. A more detailed process evaluation could have given better insight into implementation differences across schools and dose-effect relationships between implementation of specific intervention components and behavioral outcomes. A third reason for the lack of mediation effects could be that the intervention strategies aimed at improving psychosocial constructs of dietary fat intake did not work. Experimental research is needed to test the effectiveness of each of the procedures for changing hypothetical psychosocial determinants. The results of this study, for instance, suggest that the information regarding the psychosocial determinants included in the tailored feedback was ineffective and thus this information might be redundant. This highlights the need to investigate the effectiveness of a shorter tailored intervention on dietary fat intake or the need to search for new approaches to affect the psychosocial determinants of a low fat intake. At present, there is no solid evidence on the best possible ways to target psychosocial determinants of dietary fat intake in field conditions and more research is definitely required.

Fourth, our study provided some support for changes in attitudes being associated with changes in fat intake. However, none of the other psychosocial factors were reliable predictors of change in fat intake. The problem with cognitive psychosocial determinants is that they are primarily supported by cross-sectional evidence. Psychosocial determinants such as attitudes [18], self-efficacy [19] and perceived benefits [20] were identified as correlates of dietary fat intake. However, in this study, these determinants explained only less than 10% of the variance in fat intake among Flemish adolescents [21]. Furthermore, the present study showed that they were also poor predictors of change in dietary fat intake. These findings, together with the overall lack of mediation effects found in studies of adults [22]suggest that the targeted psychosocial determinants might not be relevant mechanisms of behavioral change. If this is the case, it might be inappropriate to try to change the examined cognitive psychosocial factors with respect to dietary fat intake [23]. The usefulness of the behavioral change theories we have been using to devise our interventions should then be questioned.

Finally, according to a dual-view process of environmental influences on energy-balance related behavior, the environment (in this case, aspects of the intervention) exerts both a "direct" and indirect influence on behavior [23]. The direct pathway reflects the automatic, unconscious influence of the environment on behavior manifested in the form of automatic attitude activation (e.g., thinking that it is important to regulate fat intake), unconscious behavioral mimicry (e.g., automatically mimicking the eating behavior of people that we perceive to be similar to us) and unconscious goal pursuit (e.g., environmental cues activating the goal of healthy eating). The indirect pathway reflects the mediating role of behavior-specific cognitions (such as self-efficacy) in the environment-behavior relationship. The positive intervention effects combined with the lack of mediation effects on the hypothesized mediators suggests that the intervention strategies might have had a direct impact on fat intake. It is possible that the direct pathway to health behavior change have greater saliency for adolescents than do indirect willful, cognition-mediated processes, as unintentional, irrational, impulsive and unconscious decision-making is characteristic of adolescents [24]. Developmental research has shown that brain maturation is incomplete in adolescents. When compared to adults, adolescents have lower ability to delay satisfaction, to restrain their behavior, to anticipate for future benefits and to spontaneously bring consequences into mind. Adolescents thus behave more impulsively than adults and they more often react to immediate temptations without thinking. For adolescents who engage unintentionally in healthy or unhealthy behavior the direct impact of the environment may thus have been even more influential and the cognitive processes even less important than in adults.

Although the intention of the intervention was to reduce perceived barriers in order to decrease fat intake, this mediation path had the opposite effect. Less favorable changes in perceived barriers towards a low fat diet were found in the intervention group when compared to the control group. This suppressed intervention effect on adolescents' fat intake may have arisen for two main reasons. It is possible that an intervention by measurement interactions caused this suppressed effect, i.e. that the measurement properties of the scales changed due to the intervention and repeated testing. A second explanation could be that there was an actual unexpected negative effect of the intervention on perceived barriers. It is plausible that students in the intervention group increased their awareness of the barriers associated with trying to decrease their daily fat intake.

The present results suggest that there is a need for the development of psychometrically sound instruments for the measurement of changes in theoretical psychosocial determinants of fat intake. More in-depth process evaluation measures are needed. More efforts are needed to experimentally test and compare the effectiveness of traditional and new procedures aimed at enhancing attitudes, self-efficacy, social support and perceived benefits and barriers,related to fat intake behavior. Ineffective intervention components should be identified and excluded from or modified in future interventions. More studies testing the mediating effects of the examined psychosocial constructs are needed. At present, there are no studies to compare these results with, as to our knowledge no fat intake intervention studies conducted a full mediation analysis in adolescents. Finally, more fundamental behavioral research to establish causality and test the plausibility of our theoretical variables is needed. It might be necessary to expand and modify the extant theories so that they encompass unexplored factors influencing adolescents' dietary behaviors [25].

This study has several limitations. First, all measurements were based on self-reports, that might have been affected by social desirability. Additionally, the reliability of the self-efficacy measure used in this study might have been too low to detect an intervention effect. However, previous investigations indicated that the fat intake and psychosocial measures used in this intervention were reliable and valid [8,9]. Second, the multi-component nature of the intervention made it impossible to determine which intervention components were responsible for which effects, and through which pathways. Finally, it is not clear whether weak program components or implementation issues were to blame for the lack of change in the hypothesized mediators. Although the positive effects on dietary fat intake suggest that the program was implemented as intended, the lack of extensive process evaluation measures is a limitation of the present study.

The strengths of the present study include its longitudinal randomized design, the presence of a control group, and the presence of an intervention effect on fat intake.

## Conclusion

It can be concluded that none of the examined psychosocial determinants of low fat intake were identified as reliable mediators of changes in fat intake. To draw any conclusions regarding consistent mediators of fat intake interventions in adolescent girls and regarding future effective intervention strategies, more research investigating the mediating properties of several variables is needed.

## Competing interests

The author(s) declare that they have no competing interests.

## Authors' contributions

LH performed most of the data collection and the writing of the manuscript. EC performed mediating variable analyses and wrote most of the data analytic plan and results section. IDB assisted with statistical analyses and helped to draft the manuscript. IDB, BD, LM, EC critically reviewed the manuscript for writing and intellectual content. All authors advised on study design, interpretation of data, and read and approved the manuscript.
